# Hydrops and congenital diaphragmatic hernia: reported incidence and postnatal outcomes. Analysis of the congenital diaphragmatic hernia study group registry

**DOI:** 10.1038/s41372-024-02010-5

**Published:** 2024-05-30

**Authors:** Carmen Mesas Burgos, Ashley H. Ebanks, Anna Löf-Granström, Kylie I. Holden, Anthony Johnson, Peter Conner, Matthew T. Harting, The Congenital Diaphragmatic Hernia Study Group

**Affiliations:** 1 Department of Pediatric Surgery, Karolinska University Hospital and Karolinska Institute, Stockholm, Sweden; 2 Department of Pediatric Surgery, McGovern Medical School at UT Health and Children’s Memorial Hermann Hospital, Houston, TX USA; 3 The Fetal Center, Children’s Memorial Hermann Hospital, University of Texas Health Science Center, Houston, TX USA; 4 Center for Fetal Medicine, Karolinska University Hospital and Karolinska Institute, Stockholm, Sweden

**Keywords:** Risk factors, Outcomes research

## Abstract

**Objective:**

Congenital Diaphragmatic Hernia (CDH) associated with hydrops is rare. The aim of this study was to describe the incidence of this combination of anomalies and the postnatal outcomes from a large database for CDH.

**Study design:**

Data from the multicenter, multinational database on infants with prenatally diagnosed CDH (CDHSG Registry) born from 2015 to 2021 were analyzed.

**Results:**

A total of 3985 patients were entered in the registry during the study period, 3156 were prenatally diagnosed and 88 were reported to have associated fluid in at least 1 compartment, representing 2.8% of all prenatally diagnosed CDH cases in the registry. The overall survival to discharge for CDH patients with hydrops was 43%. The hydropic CDH group had lower birth weight and gestational age at birth, and increased incidence of right-sided CDH (55%), and rate of non-repair (45%). However, the survival rate for hydropic infants with CDH undergoing surgical repair was 80%. Other associated anomalies were more common in hydropic CDH (50% vs 37%, *p* = 0.001).

**Conclusion:**

Hydropic CDH is rare, only 2.8% of all prenatally diagnosed cases, and more commonly occurring in right-sided CDH. Survival rates are low, with higher rates of non-repair. However, decision-making regarding goals of care and an aggressive surgical approach in selected cases may result in survival rates comparable to non-hydropic cases.

## Introduction

Congenital diaphragmatic hernia (CDH) is a complex and life-threatening malformation, with an incidence of 2–3/10,000 live births [1]. Overall survival rates are still low, ranging from 65 to 85% [1–4]. The prenatal detection rate with obstetrical ultrasound for CDH will vary depending on the setting but on average is ~60–80% [4, 5]. Due to the heterogeneity of this malformation, it is difficult to accurately predict outcomes in individual cases [6, 7]. Risk stratification according to several severity features allows for more adequate counseling. Prenatal factors associated with worse outcome include decreased gestational age or weight at diagnosis, decreased lung volume (observed to expected lung to head ratio (O/E LHR per US)) or total fetal lung volume (TFLV per MRI), thoracic liver location and percent herniation, thoracic stomach position, and the presence of associated structural or chromosomal anomalies [8–15].

Hydrops is a prenatal, clinical entity characterized by the presence of fluid collection in two or more body compartments, including skin or scalp edema, ascites, hydrothorax, or pericardial effusion [16–18]. Non-immune hydrops fetalis may be caused by various genetic syndromes or a consequence of hyperdynamic circulation in the fetus that leads to high-output cardiac failure [16, 18]. In the case of fetal thoracic anomalies such as CDH, hydrops may result from increased systemic venous pressure caused by obstruction of venous return to the heart. The combination of hydrops and CDH is rare, with only a few series and case reports published, and is generally associated with a worse outcome [19–21].

The objective of the current study was to increase knowledge of CDH with associated hydrops, describe the incidence of this association, and investigate postnatal outcomes using a large, multi-institutional cohort of CDH patients.

## Material and methods

Data from the Congenital Diaphragmatic Hernia Study Group (CDHSG) Registry on infants with CDH born at or transferred to any of the participating centers reporting to the registry were collected. During the specified study period, there were 83 participating centers that submitted data to the registry (Appendix 1), which includes prospectively collected information on newborns with CDH until death or discharge. The registry started in 1995. The variables collected have been updated several times and the data sheet is currently in version 5 of data collection. In 2007, when the third version was introduced, the CDH Study Group Staging System for diaphragm defect size was introduced [22] (Appendix 2), and with the fourth version in 2015, prenatal information including fetal ultrasound, magnetic resonance imaging (MRI), echocardiography, and genetic information were added. Thus, in this study, we focused on all infants reported to the CDHSG with a prenatal diagnosis between January 2015 and December 2021.

Data from patients with prenatally diagnosed CDH entered in the registry with reported fluid/edema in at least one compartment were analyzed and compared to the group of prenatally diagnosed CDH without compartmental fluid/edema. We further performed a sub-analysis of the different compartment/compartments with fluid. True hydrops were defined as fluid, edema, and/or effusions in two or more fetal compartments. Since in CDH there is a communication between the thorax and abdomen through the diaphragmatic defect, inherent to the condition, these two compartments were considered a single compartment and thus non-true hydrops. Associated variables evaluated included perinatal characteristics, number of compartments with excess of fluid, prenatal intervention, survival rates, defect size and side, associated malformations, use of extracorporeal life support (ECLS), timing of surgical repair and rates of non-repair, length of hospital stay (LOS) and need for oxygen at 30 days.

Data are presented as absolute values (*n*), percentages (%), odds ratio (OR), and 95% confidence interval (CI). Survival to discharge, need for ECLS, risk for death within the first 7 days of life, rates of non-repair, and need of oxygen at 30 days were used as endpoints in the analysis. For categorical data, Fisher’s test was performed to investigate differences between groups. For continuous variables, *t*-test or Mann–Whitney test were used. Logistic regression was used when appropriated to quantify the strengths of the association. Significance was defined as *p* ≤ 0.05. Analyses were performed using the R software version 2.38.

The CDHSG registry has been approved for use by the Committee for the Protection of Human Subjects(CPHS)/Institutional Review Board (IRB) of the McGovern Medical School at UT Health in Houston (protocol number: HSC-MS-03-223). All methods were performed in accordance with the relevant guidelines and regulations and in accordance with the Declaration of Helsinki. Informed consent for publication of the images and data was obtained.

## Results

### Fluid in at least one compartment

**Fig. 1:**
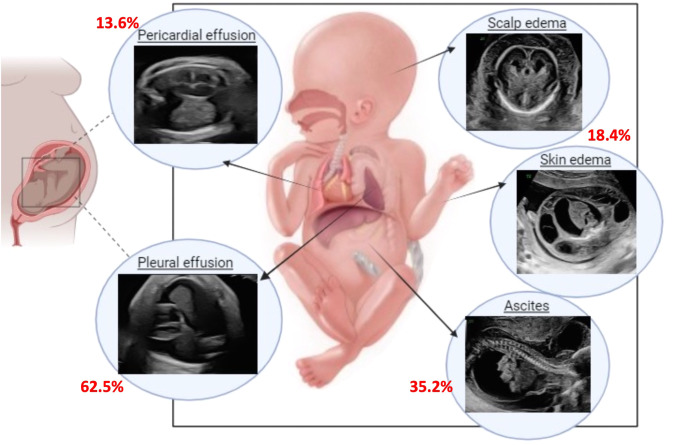
Hydrops in CDH: fluid distribution in the different compartments. Fluid distribution in the different compartments, being most common in the thorax (62.5%), followed by abdomen (35.2%), skin (18.4%) and pericardium (13.9%).

A total of 3985 patients were entered in the registry during the study period, 3156 of those were prenatally diagnosed and 88 cases were reported to also have fluid in at least one compartment, representing 2.8% of all CDH cases, with an overall survival to discharge of 43% compared to 70% for CDH cases lacking fluid in any compartment, *p* = 0.001 (Table 1, Fig. 1).

**Table 1:** Background data for the cohort of prenatally diagnosed CDH, comparing patients with reported fluid in at least 1 compartment to non-reported-hydropic.

		Fluid in compartment/compartments	Non-hydrops	*P* value
Prenatally diagnosed (*n* = 3156)		88	3068	
Sex female (%)		42.0	44.3	0.772
EGA (median [IQR])		37.00 [34.00, 38.00]	38.00 [37.00, 39.00]	<0.001
BirthWeigth (median [IQR])		2.83 [2.32, 3.22]	2.93 [2.50, 3.28]	0.103
Side (%)	Left	42.0	86.1	<0.001
	Right	55.7	12.9	
	Central	1.1	0.0	
	Bilateral	1.1	0.8	
Defect size (%)	A	2.2	8.1	0.680
	B	37.5	34.0	
	C	45.8	40.1	
	D	14.6	17.8	
O/E LHR (median [IQR])		44.95 [32.00, 52.62]	40.77 [30.84, 53.00]	0.552
Non-repairs (%)		45.5	14.7	<0.001
Type of repair (%)	Patch	66.7	63.5	0.7
ECLS (%)		34.1	30.2	0.480
Assoc anomalies (%)		50.0	37.3	0.01
Type of Assoc anomaly (%)	Cardiac	26.1	24.3	
	Chromosomal	11.4	9.6	
	Other	27.3	15.7	
	Several	18.2	11.0	
	Isolated	50.0	62.7	
O_2_ at 30D (%)		44.3	45.6	<0.001
Survival to discharge (%)		43.2	70.3	<0.001
DOL at surgery (median [IQR])		4.50 [2.00, 11.00]	4.00 [2.00, 7.00]	0.136
DOL at death (median [IQR])		1.00 [0.00, 10.75]	10.00 [1.00, 32.00]	<0.001
LOS (median [IQR])		51.00 [24.50, 93.00]	48.50 [29.00, 86.00]	0.694

*EGA* estimated gestational age, *DOL* day of life, *DC* discharge, *IQR* interquartile range, *O/E LHR* observed/expected lung to head ratio, *ECLS* extracorporeal life support.

*Significance *P* < 0.05.

There was no significant difference in median birth weight or estimated gestational age (EGA) at birth between the groups. However, prematurity (born <34 weeks EGA) was associated with higher mortality in the CDH cohort with fluid in at least one compartment (*p* = 0.027, OR 4.75, CI 1.24–20.2). The CDH group with fluid in at least one compartment had an increased incidence of right-sided CDH (55% vs 12.9%, *p* < 0.001) and higher rates of non-repairs (45.5% vs 14.7%, *p* < 0.001) (Table 1).

However, when undergoing surgical repair, survival rates for prenatally diagnosed CDH with fluid in at least one compartment was 80% (Table 2). The utilization of ECLS was similar in both groups (34% vs 30%) (Table 1), with a 36.8% survival to discharge (Table 2). The LOS was similar for CDH with fluid in at least one compartment compared to prenatally diagnosed CDH, as was the need for O_2_ at 30 days. Other associated anomalies were more common in prenatally diagnosed CDH with fluid in at least one compartment (50% vs 37%, *p* = 0.05) (Table 1).

**Table 2:** Characteristics of CDH patients who survived vs died with fluid in at least 1 compartment.

	All (*n* = 88)	Discharged alive (*n* = 38)	Died (*n* = 50)	*p* value
Hydrops with fluid at least in 2 compartments *n* (%) *(“true hydrops”)*	14 (15.9)	3 (21.4)	11 (78.6)	0.08
Hydrops with fluid in only 1 compartment or combined ascites + pleural effusion (%)	74 (84.1)	35 (47.3)	39 (52.7)	0.048
Fluid distribution in different compartments, (%):				
Ascites	10 (13.0)	4	6	
*Ascites* + *Pericardial effusion*	1 (1.3)	0	1	
*Ascites* *+* *Pericardial effusion* *+* *Pleural effusion*	2 (2.6)	0	2	
*Ascites* + *Pericardial effusion* *+* *Pleural effusion* *+* *Skin edema*	1 (1.3)	0	1	
Ascites + Pleural effusion	11 (14.3)	8	3	
*Ascites* + *Pleural effusion + Skin edema*	5 (6.5)	0	5	
*Ascites* + *Skin edema*	1 (1.3)	1	0	
Pericardial effusion	6 (7.8)	3	3	
*Pericardial effusion* *+* *Pleural effusion*	2 (2.6)	2	0	
Pleural effusion	32 (41.6)	18	14	
*Pleural effusion* + *Skin edema*	2 (2.6)	0	2	
Skin edema	4 (5.2)	1	3	
Associated anomalies *n* (%)	44 (50.0)	16 (42.1)	28 (56.0)	0.282
Type of associated anomaly *n* (%)				
Cardiac	12 (13.6)	7 (18.4)	5 (10.0)	
Chromosomal	4 (4.5)	2 (5.3)	2 (4.0)	
Dysmorphic features	1 (1.1)	0 (0.0)	1 (2.0)	
Other	11 (12.5)	1 (2.6)	10 (20.0)	
Several	16 (18.2)	6 (15.8)	10 (20.0)	
Isolated	44 (50.0)	22 (57.9)	22 (44.0)	
Side *n* (%)				
Left	37 (42.0)	9 (24.3)	28 (75.7)	0.003
Right	49 (55.7)	27 (55.1)	22 (44.9)	
Central	1 (1.1)	1 (2.6)	0 (0.0)	
Bilateral	1 (1.1)	1 (100)	0	
Non-repair (%)	40 (45.5)	0	40 (80.0)	<0.001
Surgical repair (%)	48 (54.5)	38 (80.0)	10 (20.0)	<0–001
Survival to DC (%)	38 (43.2)	38	0	
ECLS (%)	30 (34.1)	14 (36.8)	16 (32.0)	0.656

*DC* discharge, *ECLS* extracorporeal life support.

There was a higher rate of associated anomalies among CDH patients with fluids in at least one compartment who died before discharge (56 vs 42%) (Table 2).

### True hydrops

The distribution of fluid collection in the different compartments is summarized in Table 3 and Fig. 1. Only 14 patients had fluid collections in more than one compartment (excluding the combination of pleural effusion and ascites for reasons noted above), representing the “true” hydrops cohort with an incidence of only 0.35%, and having a high mortality rate of 79%.

**Table 3:** Sub-analysis of true vs non-true hydrops.

		Fluid in >1 compartment	Fluid in 1 compartment (or combined ascites + pleural effusion)	*P* value
*n*		14	74	
Side (%)	Left	3 (21.4)	34 (45.9)	<0.001
	Right	11 (78.6)	38 (51.4)	
	Central	0 (0.0)	1 (1.4)	
	Bilateral	0 (0.0)	1 (1.4)	
Repair done (%)		7 (50.0)	41 (55.4)	0.775
Patch repair (%)		5 (71.4)	27 (65.9)	1.000
ECLS (%)		6 (42.9)	24 (32.4)	0.542
Associated anomalies (%)		4 (28.6)	40 (54.1)	0.143
Type of associated anomaly (%)	Cardiac	1 (7.1)	11 (14.9)	0.489
	Chromosomal	0 (0.0)	4 (5.4)	
	Dysmorphic features	0 (0.0)	1 (1.4)	
	Other	0 (0.0)	11 (14.9)	
	More than 1	3 (21.4)	13 (17.6)	
	Isolated	10 (71.4)	34 (45.9)	
O_2_ requirement at 30 DOL (%)		5 (100.0)	34 (72.3)	0.314
Survival to discharge (%)		3 (21.4)	35 (47.3)	0.086
DOL at surgery (median [IQR])		4.00 [2.00, 8.50]	5.00 [3.00, 11.00]	0.617
DOL at death (median [IQR])		1.00 [0.00, 23.00]	1.00 [0.50, 8.50]	0.895
DOL at DC (median [IQR])		67.00 [46.50, 221.00]	50.00 [22.00, 93.00]	0.433

*DOL* day of life, *DC* discharge, *IQR* interquartile range, *ECLS* extracorporeal life support.

*P < 0.05.

In the group of true hydrops (fluid in more than one compartment), there was a high rate of right-sided CDH (78.6%), skin edema was present in 64.3%, surgical repair was done in 50% of the cases with survival to discharge in the surgically treated group of 43%. Only 28.7% of true hydropic CDH had associated anomalies compared to the group of “non-true hydrops” (54.1%) (Table 3).

In the event of isolated skin edema, there is a higher mortality risk before discharge (OR 5.08, 95% CI 1.25–34.3).

### Laterality of the defect

Left-sided CDH with fluid in at least one compartment had lower survival rates than right-sided CDH with fluid in at least one compartment (24 vs 55%, OR = 3.82, *p* = 0.005), and higher rates of associated anomalies (67.6 vs 34.7, *p* = 0.004) (Table 4).

**Table 4:** Sub-analysis of the laterality of the defect (left to right side only).

	Left (*n* = 37)	Right (*n* = 49)	*p*-value
EGA (median [IQR])	37.00 [34.00, 38.00]	37.00 [34.00, 38.00]	0.654
FETO *n* (%)	1 (2.7)	4 (8.2)	0.3
Non-repairs (%)	75.7	24.5	<0.001
Patch repair (%)	66.7	64.9	1.000
ECLS (%)	16.2	44.9	0.006
Associated anomalies (%)	67.6	34.7	0.004
Type of associated anomaly (%)	10.8	14.3	0.014
Cardiac	8.1	2.0	
Chromosomal	18.9	8.2	
Other	27.0	10.2	
Several	32.4	65.3	
Isolated			
O_2_ att 30 DOL (%)	53.8	81.1	0.073
Survival to discharge (%)	24.3	55.1	0.005
DOL at surgery (median [IQR])	7.00 [4.00, 11.00]	4.00 [2.00, 9.00]	0.387
DOL at death (median [IQR])	1.00 [0.00, 3.25]	3.00 [1.00, 34.50]	0.043
LOS (median [IQR])	31.00 [3.25, 57.25]	59.00 [30.00, 95.00]	0.071

EGA: estimated gestatioal age, *DC* discharge, *ECLS* extracorporeal life support, *DOL* day of life, *IQR* interquartile range, *FETO* fetoscopic endotracheal occlusion.

The rates of non-repairs were higher in the left-sided CDH with fluids in at least one compartment compared to right sided (75% vs 24.5%, *p* < 0.0001) (Table 2).

## Discussion

In this prospective multicenter study, we conclude that hydropic CDH is rare, only 2.8% of all prenatally diagnosed cases, and more commonly occurring in right-sided CDH. Survival rates are low, with higher rates of non-repair. Hydrops is a clinical entity characterized by the presence of skin and scalp edema or fluid in two or more body compartments. In cases of fetal thoracic anomalies, such as CDH, it is thought to be caused by obstruction of venous return to the heart leading to elevated systemic venous pressures [23].

When using the term ‘hydrops’ in CDH, effusions in the thoracic cavity and abdominal cavity concomitant to the side of the defect, should be considered as one compartment for obvious reasons, avoiding confusion. Thus, true hydrops in CDH should refer to patients with excess fluid in at least two non-communicating compartments.

However, the presence of excess fluid in only one compartment is associated with worse outcome. Even if the association is rare, nearly 3% of all prenatally diagnosed CDH will present with fluid in one compartment, the survival rates are low (47.3%, Table 3). True hydropic CDH is even rarer, only identified in 0.35% of the prenatally diagnosed CDH cases, and more commonly occurring in right-sided CDH. Survival rates for true hydropic CDH are very low: 78% of patients with true hydrops did not survive to discharge.

Earlier studies have reported a 46% survival rate for hydropic fetuses and as high as 90% mortality rate for hydrops associated with CDH [24].

Malformations are present in half of the CDH with fluid/edema, true hydrops (fluid in at least two compartments), or non-true hydrops (fluid in 1 compartment), and more common among those who died before discharge (56% vs 42%, Table 2).

Skin edema seems to be an independent risk factor for worse outcome, with higher mortality. However, even though the pathophysiological mechanisms underlying this event remain unknown, an increased central venous pressure and/or obstructed lymphatic drainage may be the most plausible explanation [25].

Although the pathogenesis of hydrops in fetuses with CDH is poorly understood, right-sided lesions seem to be more likely to cause hydrops in CDH. Most of the cases presented here were right sided and/or had a major portion of the liver in the chest, thus the pathophysiology may be similar to other intrathoracic lesions, with mediastinal obstruction from solid organ herniation obstructing blood return to the heart. Since there is a confined space within the thorax, the herniation of a solid organ such as the liver, leaving a smaller portion intraabdominally, may lead to kinking and obstruction of the inferior vena cava, portal, or hepatic vein [26]. There are a few cases reports in the literature with the successful placement of thoraco-amniotic shunt prenatally [27].

Hydrops is more commonly associated with right-sided CDH, as many as half of the patients with hydrops have a right-sided defect, but left-sided CDH with associated hydrops has a poorer outcome, with higher rates of associated anomalies, high rates of non-repairs, and is overrepresented among children who died before discharge (75% of the left-sided CDH with hydrops will demise, probably due to the complexity of the CDH). In the absence of fetal hydrops, left-sided CDH has usually been reported to have better survival rates [28, 29].

Reasons for non-repair are the critical condition of the newborn, due to the severity of the hernia and impossibility to stabilized, the co-occurrence of other major associated anomalies or clinical deterioration non-reversible, upon parental request or the multidisciplinary teams best judgment. Despite high rates of non-repairs, good patient selection and a more aggressive surgical approach can lead to acceptable survival rates: 80% of the patients repaired survive to discharge. ECLS support was offered to the same extent to patients who survived and those hydrops patients who died before discharge.

Some of the limitations of this study is that the variable reported to the registry is limited and varying over time. Some of the parameters, e.g., hernia size and O/E LHR, were introduced in recent most versions of the CDHSG reporting form. The rate of prenatal diagnosis has increased over time and the availability for genetic testing has also changed substantially during the period the data were collected. Management protocols have evolved [30, 31] and decision-making by multidisciplinary team approach has become more standard of care in recent years. Also, the interpretation of the results cannot be universally generalized to the entire CDH population, since the data only includes patients born at or transferred to the participating centers. Another limitation if the lack of long-term follow-up data, since the data collection only comprises the time of in-hospital stay, and therefore does not consider any events occurring after the patient is discharged from the center.

In conclusion, the association of CDH and hydrops in the fetus is rare and the sole presence of excess fluid or edema in 1 compartment is associated with poorer outcome. The combination is associated with the presence of other associated anomalies and right-sided defects with liver involvement. Special attention should be given to the presence of skin edema resulting in higher mortality rates. An initially aggressive surgical approach may result in acceptable survival rates comparable to non-hydropic cases. Early decision-making regarding the aims of care clearly influence outcome, since survival varies depending on the severity of the CDH and the presence of other associated anomalies.

## Supplementary information

Legend to supplementary material

Contributing Centers

CDHSG Staging

## Data Availability

The datasets generated during and/or analysed during the current study are not publicly available due GDPR regulations but are available from the corresponding author on reasonable request.
