# Supplementary material for: Hydrops and congenital diaphragmatic hernia: reported incidence and postnatal outcomes. Analysis of the congenital diaphragmatic hernia study group registry
Source: J Perinatol. 2024 May 30;44(9):1340–6. doi: 10.1038/s41372-024-02010-5 (PMC11379622; doi:10.1038/s41372-024-02010-5)
Supplement: Supplementary file 1 — Legend to supplementary material [file 41372_2024_2010_MOESM1_ESM.docx]

**Supplementary material Legends**

**Appendix 1: Centers contributing to the CDH Study Group**

**Appendix 2: CDHSG Size Classification**
