# Supplementary material for: Hydrops and congenital diaphragmatic hernia: reported incidence and postnatal outcomes. Analysis of the congenital diaphragmatic hernia study group registry
Source: J Perinatol. 2024 May 30;44(9):1340–6. doi: 10.1038/s41372-024-02010-5 (PMC11379622; doi:10.1038/s41372-024-02010-5)
Supplement: Supplementary file 2 — Contributing Centers [file 41372_2024_2010_MOESM2_ESM.docx]

| Hosp | City | StateProv | Country |
| --- | --- | --- | --- |
| Alberta Children's Hospital | Calgary | AB | Canada |
| Arkansas Children's Hospital | Little Rock | AR |  |
| Astrid Lindgren Children's Hospital | Stockholm |  | Sweden |
| Azienda Ospedaliera Papa Giovanni XXIII | Bergamo |  | Italy |
| BC Children's & Women's Health Centre | Vancouver | BC | Canada |
| Cairo University Pediatric Hospital (Aboul Reesh) | Cairo |  | Egypt |
| Carolinas Medical Center, Levine Children's Hospital | Charlotte | NC |  |
| Children's Hospital & Research Center Oakland | Oakland | CA |  |
| Childrens Hospital at Skanes University Hospital | Lund |  | Sweden |
| Children's Hospital Boston | Boston | MA |  |
| Children's Hospital of Akron | Akron | OH |  |
| Children's Hospital of Georgia - AU Health | Augusta | GA |  |
| Children's Hospital of Illinois at OSF St. Francis Med Center | Peoria | IL |  |
| Children's Hospital of Los Angeles | Los Angeles | CA |  |
| Childrens' Hospital of Orange County | Orange | CA |  |
| Children's Hospital of San Antonio | San Antonio | TX |  |
| Children's Hospital of Wisconsin | Milwaukee | WI |  |
| Children's Hospital Omaha | Omaha | NE |  |
| Childrens Hospital, University Bonn | Bonn |  | Germany |
| Children's Hospitals and Clinics (Minneapolis) | Minneapolis | MN |  |
| Children's Memorial Hermann Hospital | Houston | TX |  |
| Children's of Alabama | Birmingham | AL |  |
| Cincinnati Children's Hospital Medical Center | Cincinnati | OH |  |
| Cleveland Clinic Foundation- Children's Hospital | Cleveland | OH |  |
| Connecticut Children's Medical Center | Hartford | CT |  |
| Dell Children’s Medical Center of Central Texas | Austin | TX |  |
| Duke University Medical Center | Durham | NC |  |
| Emory University | Atlanta | GA |  |
| Golisano Children’s Hospital at Strong | Rochester | NY |  |
| Hospital Clinico Universidad Católica de Chile | Santiago | RM | Chile |
| IRCCS Fondazione Ca' Granda Ospedale Maggiore Policlinico | Milano |  | Italy |
| James Whitcomb Riley Children's Hospital | Indianapolis | IN |  |
| Johns Hopkins All Children’s Hospital | St Petersburg | FL |  |
| Johns Hopkins Hospital | Baltimore | MD |  |
| Juan P. Garrahan Children Hospital | Buenos Aires |  | Argentina |
| La Paz University Hospital | Madrid |  | Spain |
| Le Bonheur Children’s Medical Center | Memphis | TN |  |
| Legacy Emanuel Children's Hospital | Portland | OR |  |
| Loma Linda University Children's Hospital | Loma Linda | CA |  |
| Lucile Salter Packard Children's Hospital | Palo Alto | CA |  |
| Mattel Children's Hospital at UCLA | Los Angeles | CA |  |
| Miami Valley Hospital | Dayton | OH |  |
| National Center for Child Health and Development | Tokyo |  | Japan |
| NICU Health Sciences Centre | Winnipeg | MB | Canada |
| Norton Children's Hospital | Louisville | KY |  |
| Osaka University Hospital | Suita-shi | Osaka | Japan |
| Ospedale Pediatrico Bambino Gesù | Rome |  | Italy |
| Palmetto Health Richland | Columbia | SC |  |
| Phoenix Children's Hospital | Phoenix | AZ |  |
| Polish Mother's Memorial Hospital Research Institute | Lodz |  | Poland |
| Primary Children's Hospital | Salt Lake City | UT |  |
| Radboud University Nijmegen Medical Centre | Nijmegen |  | The Netherlands |
| Rady Children's Hospital | San Diego | CA |  |
| Research Center for Obstetrics, Gynecology and Perinatology | Moscow |  | Russia |
| Research Institute at Nationwide Children’s Hospital | Columbus | OH |  |
| Royal Children's Hospital | Parkville | Victoria | Australia |
| Royal Hospital for Sick Children | Glasgow |  | Scotland |
| Shands Children's Hospital/University of Florida | Gainesville | FL |  |
| Sophia Children's Hospital | Rotterdam |  | The Netherlands |
| St. Francis Children's Hospital | Tulsa | OK |  |
| St. Joseph's Hospital and Medical Center | Phoenix | AZ |  |
| St. Louis Children's Hospital | St. Louis | MO |  |
| St. Louis Univ School of Medicine at SSM Health Cardinal Glennon Children's Hospital | St. Louis | MO |  |
| Stollery Children's Hospital | Edmonton | AB | Canada |
| Sydney Children's Hospital | Randwick | NSW | Australia |
| Texas Children's Hospital | Houston | TX |  |
| The Children's Hospital at OU Medical Center | Oklahoma City | OK |  |
| The Children's Hospital of Pittsburgh of UPMC | Pittsburgh | PA |  |
| The Hospital for Sick Children | Toronto | Ontario | Canada |
| The Queen Silvia Children’s Hospital SU/Östra | Gothenburg |  | Sweden |
| Tufts Medical Center | Boston | MA |  |
| UNC School of Medicine | Chapel Hill | NC |  |
| University Childrens Hospital | Uppsala |  | Sweden |
| University Malaya Medical Centre | Kuala Lumpur |  | Malaysia |
| University of Michigan, C.S. Mott Children's Hospital | Ann Arbor | MI |  |
| University of Nebraska Medical Center | Omaha | NE |  |
| University of Padua | Padua |  | Italy |
| University of Texas Medical Branch at Galveston | Galveston | TX |  |
| University of Virginia Medical School | Charlottesville | VA |  |
| Vanderbilt Children's Hospital | Nashville | TN |  |
| Vladivostok State Medical University | Vladivostok |  | Russia |
| Winnie Palmer Hospital for Women & Babies | Orlando | FL |  |
| Yale New Haven Children's Hospital | New Haven | CT |  |
