# Supplementary figures and images for: Hydrops and congenital diaphragmatic hernia: reported incidence and postnatal outcomes. Analysis of the congenital diaphragmatic hernia study group registry
Source: J Perinatol. 2024 May 30;44(9):1340–6. doi: 10.1038/s41372-024-02010-5 (PMC11379622; doi:10.1038/s41372-024-02010-5)

Defect A

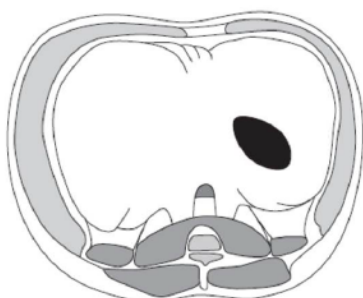

Defect B

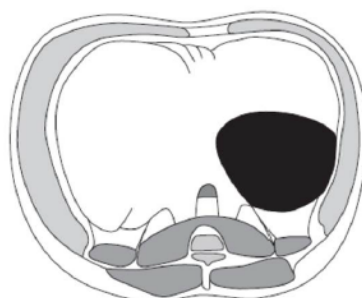

Defect C

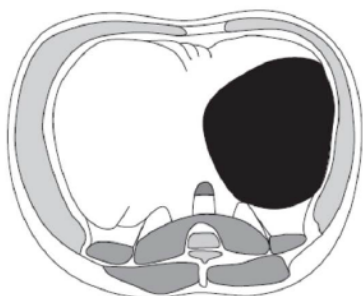

Defect D

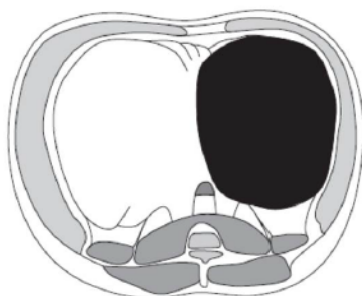

Supplement: Supplementary file 3 — CDHSG Staging [file 41372_2024_2010_MOESM3_ESM.pdf]
